# 3,5‐Dicaffeoylquinic Acid Delayed Aging and Promoted Oxidative Stress Tolerance via Activation of the SKN‐1/Nrf2 Signaling Pathway

**DOI:** 10.1002/fsn3.71532

**Published:** 2026-02-17

**Authors:** Rong Li, Mingfang Tao, Jinzhan Yuan, Yechuan Huang, Tingting Xu, Xiaoyun Xu

**Affiliations:** ^1^ Characteristic Food Function Mining and Comprehensive Utilization Research Center Jingchu University of Technology Jingmen China; ^2^ Hubei Key Laboratory of Nutritional Quality and Safety of Agro‐Products, Institute of Agricultural Quality Standards and Detection Technology Hubei Academy of Agricultural Sciences Wuhan China; ^3^ College of Plant Science and Technology Huazhong Agricultural University Wuhan China; ^4^ Key Laboratory of Environment Correlative Dietology (Ministry of Education), College of Food Science and Technology Huazhong Agricultural University Wuhan China

**Keywords:** 3,5‐dicaffeoylquinic acid, anti‐aging, *Caenorhabditis elegans*, oxidative stress tolerance, SKN‐1/Nrf2

## Abstract

3,5‐Dicaffeoylquinic acid (3,5‐diCQA), as a plant‐derived polyphenol, exhibits multiple bioactivities, including anti‐inflammation, antioxidation, and anti‐diabetes. A previous report demonstrated that 3,5‐diCQA increased the lifespan and promoted the healthspan in *Caenorhabditis elegans* . Nevertheless, the molecular mechanisms underlying the function of 3,5‐diCQA remain to be further determined. In this study, 3,5‐diCQA promoted the transfer of SKN‐1 to nucleus and upregulated the expressions of its downstream genes. Moreover, 3,5‐diCQA enhanced oxidative stress tolerance and decreased ROS level in a *skn‐1*‐dependent manner. Consistently, 3,5‐diCQA remarkably reduced the ROS level and delayed senescence of MRC‐5 cells by activating Nrf2. Notably, molecular docking results revealed that 3,5‐diCQA was found to occupy the binding pocket of Keap 1 (Kelch‐like epichlorohydrin‐associated protein 1), a cytoplasmic repressor of Nrf2, thereby promoting Nrf2 activation. Overall, this study demonstrated that SKN‐1/Nrf2 signaling is essential for 3,5‐diCQA to exert its anti‐aging and stress resistance‐enhancing effects. Our findings elucidate novel mechanisms by which 3,5‐diCQA activates the SKN‐1/Nrf2 pathway, highlighting its promise as candidate for delaying aging and attenuating oxidative stress‐related disorders.

## Graphical abstract

3,5‐Dicaffeoylquinic acid promoted lifespan and stress tolerance capability of *C. elegans* in a *skn‐1*‐dependent manner. 3,5‐Dicaffeoylquinic acid reduced the ROS level and delayed senescence of MRC‐5 cells by activating Nrf2. 3,5‐Dicaffeoylquinic acid directly binded to the pocket of Kelch‐like epichlorohydrin‐associated protein 1 (Keap1) to facilitate Nrf2 activation.

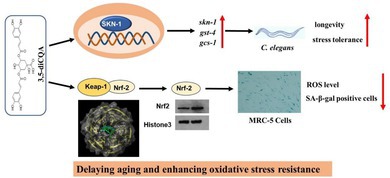

## 1 Introduction

Aging is a kind of time‐related functional decline, leading to gradual degradation of various physiological functions and increasing mortality risk (Harman 2004). As a key contributor to various chronic conditions, modulating the aging process holds significant potential in preventing age‐related pathologies and promoting health (Niccoli and Partridge 2012). Oxidative stress resilience serves as a fundamental mechanism for multicellular organisms to increase environmental adaptation and delay senescence (Peng et al. 2014). Nuclear factor erythroid‐derived 2‐related factor 2 (Nrf2), the mammalian counterpart of *Caenorhabditis elegans* ( *C. elegans* ) SKN‐1, plays a pivotal role in protecting cells against oxidative damage (Blackwell et al. 2015; Laura et al. 2025). Notably, numerous natural compounds have been documented to possess anti‐aging and antioxidant properties by utilizing models ranging from cells to animals and humans (Gurău et al. 2018; Mária and Ingrid 2017; Vaiserman et al. 2016; Zhou et al. 2024). Consequently, activators of Nrf2/SKN‐1 could serve as promising candidates for scavenging reactive oxygen species (ROS) and enhancing antioxidant capacities, thereby delaying senescence and improving quality of life.

Under normal physiological conditions, Nrf2 is retained in the cytoplasm via direct binding to its suppressor Keap1, followed by ubiquitination and rapid proteasomal degradation (Qin and Hou 2016). When stimulated by certain bioactive compounds, Nrf2 evades repression by Keap1 and transfers into the cell nucleus, thus activating the expression of phase II detoxification enzyme genes including superoxide dismutase, catalase, and glutathione transferase to regulate cellular antioxidant responses (Nguyen et al. 2009; Schmidlin et al. 2019; Tong et al. 2006). In *C. elegans* , the phase II defense response is orchestrated by SKN‐1, a critical transcription factor (Tullet et al. 2008). The expression of SKN‐1 in the intestine mediates stress resistance, whereby its nuclear translocation triggers the expression of antioxidant genes in response to stress (An and Blackwell 2003). Notably, SKN‐1/Nrf2 is a key factor for longevity regulation, with well‐documented studies indicating that moderate overexpression of SKN‐1/Nrf2 promotes longevity in nematodes and *Drosophila*, whereas *skn‐1* deficiency leads to lifespan shortening in worms (Lapierre and Hansen 2012; Sykiotis and Bohmann 2008). The aging process leads to a gradual decline in SKN‐1/Nrf2 activity, which is coupled with diminished expression and impaired activation of its downstream genes under acute oxidative stress (Rahman et al. 2013). Thus, SKN‐1/Nrf2 could serve as a pivotal intervention target that mediates oxidative stress responses and delays aging progression.

Chlorogenic acids (CGAs), a significant class of bioactive dietary polyphenols present in fruits and plants, including coffee, tea, and many traditional Chinese medicines, exhibit anti‐inflammatory, antioxidant, and neuroprotective activities (Clifford et al. 2017). 3,5‐diCQA, namely isochlorogenic acid A, is a predominant derivative of chlorogenic acid (CGA) and exhibits faster absorption and more efficient metabolism compared to other CGAs (Farrell et al. 2011). Pharmacological investigation has found that 3,5‐diCQA ameliorates oxidative damage in Caco‐2 cells through Nrf2 activation (Liang and Kitts 2018). Additionally, our previous report has demonstrated that 3,5‐diCQA prolonged the survival time of *C. elegans* via inhibiting the insulin/insulin‐like growth factor signaling (IIS) pathway (Li et al. 2021). SKN‐1 is involved in promoting longevity in organisms with diminished IIS signaling. Specifically, downregulation of the IIS pathway induces SKN‐1 to translocate to the nucleus in the intestine, leading to the activation of conserved phase II detoxification genes (Tullet et al. 2008). Therefore, whether the SKN‐1/Nrf2 signaling pathway has a positive effect on the anti‐aging function of 3,5‐diCQA remains to be further confirmed.

In this work, we investigated the role of SKN‐1/Nrf2 in the mechanisms by which 3,5‐diCQA promotes lifespan and oxidative stress tolerance using a *C. elegans* model. Moreover, MRC‐5 cells model combined with molecular docking analysis were applied to explore the regulatory effects of 3,5‐diCQA on Nrf2 and to identify its potential binding sites. This study provides a valuable reference that natural compounds capable of activating the SKN‐1/Nrf2 may serve as effective agents to delay senescence and prevent oxidative stress‐related diseases.

## 2 Materials and Methods

### 2.1 Reagents

3,5‐diCQA (≥ 98%), fluorodeoxyuridine, dimethyl sulfoxide (DMSO), 2′,7′‐dichlorodihydro fluorescein diacetate (H_2_DCF‐DA), and hydrogen peroxide (> 30%, w/w) were obtained from our previous study (Li et al. 2021). 3,5‐diCQA was dissolved in DMSO to obtain a 100 mM stock solution and stored in a refrigerator at −80°C.

### 2.2 Strains and Maintenance Conditions

The nematodes and *Escherichia coli* OP50 ( *E. coli* OP50) utilized in this work were ordered from Caenorhabditis Genetics Center (CGC) (University of Minnesota, USA), including Bristol (wild‐type, N2), CL2166 [(*pAF15*) *gst‐4p::GFP::NLS*], EU1[*skn‐1*(*zu67*)*IV*], and LD1 ldIs7[*skn‐1b/c::GFP + rol‐6* (*su1006*)*]*. All strains were cultured at 20°C on nematode growth medium (NGM) plates supplemented with *E. coli* OP50 as food resource.

### 2.3 Oxidative Stress Resistance Assay

The L4‐stage larvae of N2 or EU1 were inoculated into a 96‐well plate adding S‐complete substrate supplemented with 150 μM FudR, heat‐killed *E. coli* OP50 (65°C, 30 min), and different doses of 3,5‐dicaffeoylquinic acid (25, 50, and 100 μM) (treatment groups), with S‐complete substrate +0.1% DMSO used as the control group. On the 7th day post culture, the nematodes were inoculated into 1 mM H_2_O_2_ plates for oxidative stress experiments at 20°C (Wang et al. 2024). The survivals of worms were counted every 2 h till all of them died.

### 2.4 Intracellular ROS Measurement

The *C. elegans* was cultured according to the above methods. On the 6th day of adulthood, the nematodes were transferred into a black 96‐well plate added with 50 μM H_2_DCF‐DA solution, 30 worms per well (Xiong et al. 2018). A fluorescence microplate reader (Thermo Labsystems, Waltham, USA) was used to record the fluorescence signal every 20 min for 2 h.

### 2.5 SKN‐1::GFP Subcellular Localization Determination

L4‐stage LD1 larvae were incubated with 3,5‐diCQA (50 μM) or DMSO (0.1%) for 2 h, followed by fluorescence microscopy (Olympus, Japan) imaging to determine SKN‐1::GFP localization. The nuclear translocation patterns of SKN‐1::GFP were indicated by the percentage of its locations in the cytoplasm, intermediate, and nucleus (Tao et al. 2023).

### 2.6 Fluorescence Measurement of GFP Protein

Synchronized L1‐stage larvae of CL2166 strains (a GFP reporter for GST‐4) were incubated with 3,5‐diCQA (50 μM) or DMSO (0.1%) for 3 days and then anesthetized with 10 mM sodium azide (Tang et al. 2023). Subsequently, the fluorescence intensity of the worm bodies was photographed.

### 2.7 Quantitative RT‐PCR Analysis

Worms were cultured according to the protocols outlined in section 2.3, and adult nematodes were collected on the 3rd day. Total RNA was extracted and synthesized to cDNA with cDNA synthesis Kit (Vazyme Biotech, China). The qRT‐PCR reactions were conducted following the protocols described in a previous report (Wang et al. 2024; Ban et al. 2021). Gene expression was calculated by the 2^−ΔΔCT^ method with *act‐1* used as an endogenous control. The gene primer sequences can be found in Table S1.

### 2.8 Molecular Docking Between 3,5‐Dicaffeoylquinic Acid and Keap1

Molecular docking was carried out to investigate the binding of ligands to Keap1 using Schrödinger package (2018). Keap1 crystal structure and molecular structure of positive ligand 4‐methoxy‐phenylsulfonamide were downloaded from the RSCB PDB database (PDB ID: 4IQK) (Marcotte et al. 2013; Satoh et al. 2015). The molecular structure of 3,5‐diCQA was drawn and energy‐minimized before being docked into the Keap1 protein's binding grids in standard precision mode. The docking score was employed to evaluate the binding affinity between the ligands and the protein.

### 2.9 Cell Culture and MTT Assay

MRC‐5 human lung fibroblasts (MRC‐5 cells) were sourced from the China Typical Culture Preservation Center Cell bank (Wuhan University, China). MRC‐5 cells were maintained in MEM supplemented with 10% fetal bovine serum (FBS), 100 U/mL penicillin, and 100 μg/mL streptomycin, under normal culture conditions (37°C, 5% CO₂, humidified environment). For cell viability determination, cells were plated in 96‐well plates at a density of 1 × 10^4^ cells per well and exposed to 0.1% DMSO along with varying doses of 3,5‐diCQA for 24 h. MTT (5 mg/mL) was incubated with the cells for 4 h before measuring the absorbance at 490 nm.

### 2.10 ROS, SA‐β‐Gal Staining, and Protein Expression Assays

For ROS and SA‐β‐Gal staining detections, MRC‐5 cells were incubated in 24‐well plates at a density of 1 × 10^5^ cells per well and exposed to 0.1% DMSO along with multiple dosage levels of 3,5‐diCQA applied for 24 h. The cells were collected to measure the ROS level and SA‐β‐galactosidase activity with corresponding kits (Beyotime Biotechnology, Shanghai, China). In order to analyze protein expression, cells (4 × 10^5^ cells/well) were exposed to DMSO (0.1%) and 3,5‐diCQA (50 μM) for 24 h. Nuclear and cytoplasmic protein extracts were obtained using commercial extraction kits to measure the expressions of Keap1 and Nrf2 protein based on the manufacturer's recommendations (Biofavor Biotechnology Company, Hubei, China).

### 2.11 Statistical Analysis

GraphPad Prism 7.0 (La Jolla, CA, USA) was employed for lifespan analysis. For multiple comparisons, one‐way analysis of variance (ANOVA) followed by Duncan's test and student's *t*‐test were used. A *p* < 0.05 was considered statistically significant.

## 3 Results

### 3.1 3,5‐diCQA Reduced ROS Levels and Improved Oxidative Stress Tolerance of *C. elegans*

**FIGURE 1:**
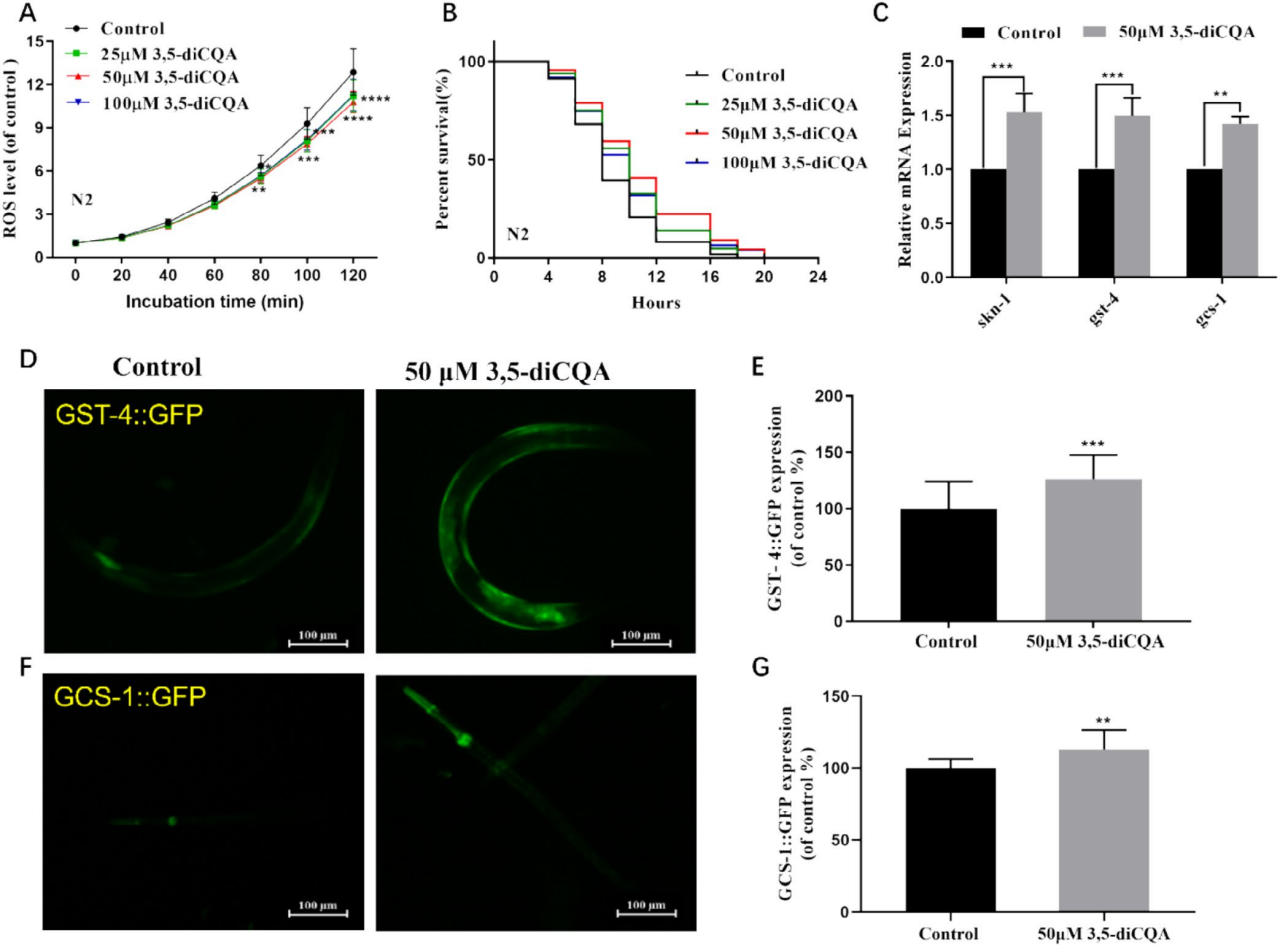
3,5‐diCQA improved oxidative stress tolerance and upregulated the expressions of stress‐related genes and proteins in *C. elegans* . (A) 3,5‐diCQA decreased the intracellular ROS level of N2 worms. (B) Survival curves of wild‐type worms under oxidative stress. (C) The expression levels of *skn‐1*, *gst‐4*, and *gcs‐1* of wild‐type worms. (D) Fluorescent images and (E) determination of GST‐4::GFP expression in CL2166 strains. (F) Fluorescent images and (G) determination of GCS‐1::GFP expression in LD1171 strains. Data were displayed as mean ± SD. **p < 0.01, ***p < 0.001, ****p < 0.0001.

Overproduction of ROS exerts detrimental effects on aging and stress resistance, whereas supplementation with natural antioxidants can scavenge free radicals, thereby slowing the aging process (Finkel and Holbrook 2000; Liochev 2013). As shown in Figure 1A, supplementation with 3,5‐diCQA obviously decreased the intracellular ROS levels in wild‐type nematodes compared to the control group (*p* < 0.05). Moreover, treatment with 3,5‐diCQA at doses of 25, 50, and 100 μM remarkably prolonged the mean survival time of wild‐type nematodes exposed to oxidative stress by 12.11%, 21.71%, and 11.66%, respectively (*p* < 0.01, Figure 1B, Table 1). Based on its optimal lifespan‐extending efficacy, 50 μM 3,5‐diCQA was employed in subsequent experiments. Moreover, the expressions of *skn‐1* and its downstream genes, *gst‐4* and *gcs‐1*, were markedly increased in nematodes exposed to 3,5‐diCQA (Figure 1C, *p* < 0.05). Notably, treatment with 3,5‐diCQA consistently increased the expression of GST‐4::GFP and GCS‐1::GFP by 26.24% and 13.25% in the CL2166 and LD1171 strains, respectively (*p* < 0.001 and *p* < 0.01, Figure 1D–G). These findings indicated that 3,5‐diCQA significantly enhanced the antioxidant capacity of *C. elegans* .

**TABLE 1:** The effect of 3,5‐diCQA on the oxidative stress resistance of N2 worms.

Treatments	Mean lifespan (hours ± SEM)	Percentage change	Number of worms	*p* value
Control	8.75 ± 0.26^a^	—	160	—
25 μM 3,5‐diCQA	9.81 ± 0.28^b^	12.11%	165	0.0044
50 μM 3,5‐diCQA	10.65 ± 0.34^c^	21.71%	157	< 0.0001
100 μM 3,5‐diCQA	9.77 ± 0.31^bc^	11.66%	158	0.0053

*Note:* Different letters indicated a significant difference between the two groups and *p* values were analyzed by the log‐rank test.

### 3.2 3,5‐diCQA Prolonged the Lifespan of *C. elegans* by Activating SKN‐1

**FIGURE 2:**
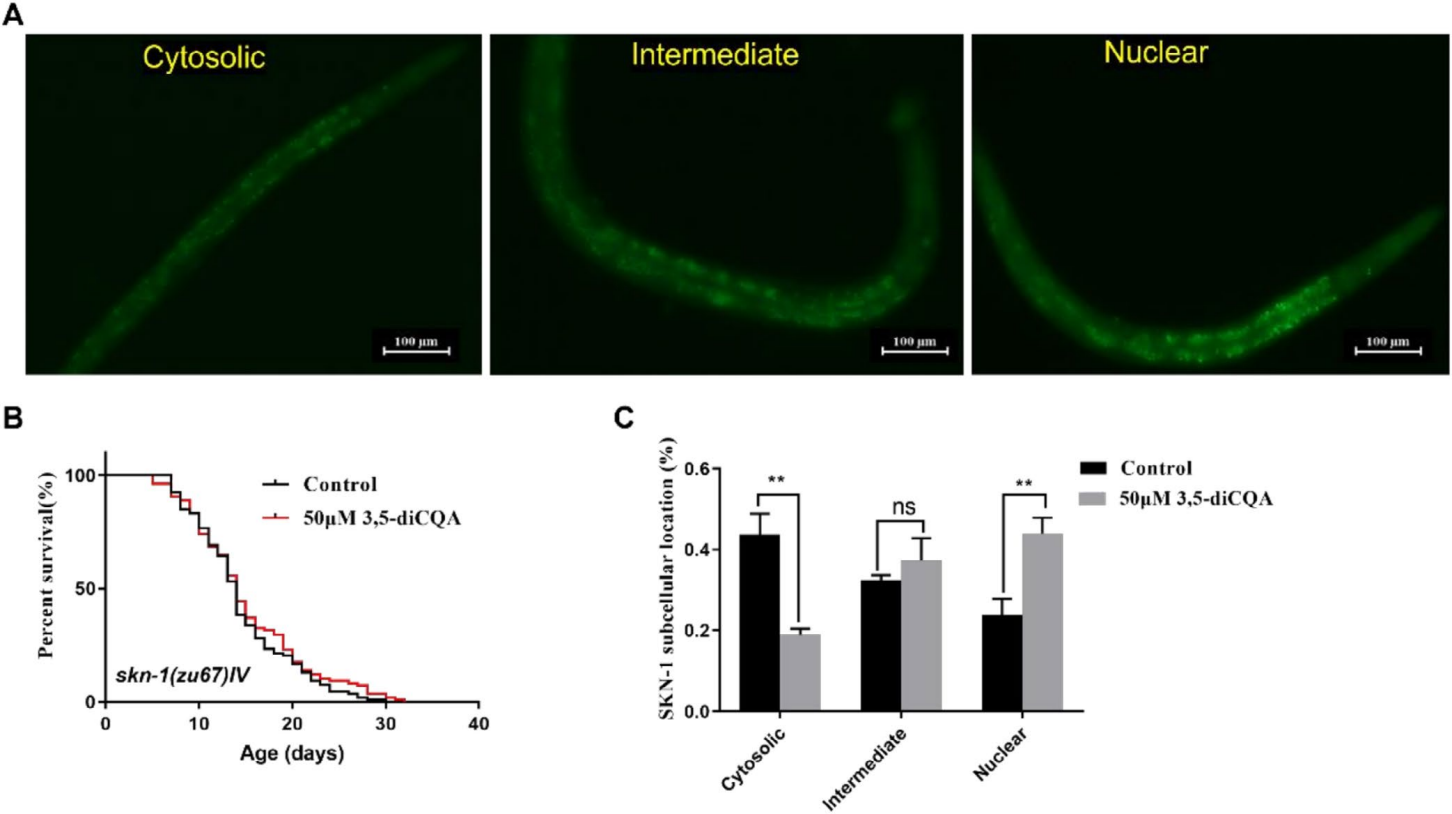
3,5‐diCQA prolonged the lifespan of *C. elegans* by activating SKN‐1. (A) Typical images of SKN‐1::GFP localization in transgenic strain LD1: Cytosolic, intermediate, and nuclear. (B) Survival curves of *skn‐1*(*zu67*) mutants. (C) Quantification of SKN‐1::GFP localization. Data were displayed as mean ± SD. **p < 0.01; ns. no significance.

The transcription factor SKN‐1 has been found to promote longevity of *C. elegans* by mediating the oxidative stress response pathway (Blackwell et al. 2015). Thus, the function of SKN‐1 in 3,5‐diCQA‐mediated longevity was investigated. In our earlier report, 3,5‐diCQA was found to prolong the survival time of wild‐type nematodes (Li et al. 2021). However, deletion of *skn‐1* eliminated the pro‐longevity effect induced by 3,5‐diCQA, illustrating that 3,5‐diCQA‐mediated lifespan extension relied on *skn‐1* (*p* > 0.05, Figure 2B, Table S2). Furthermore, the nuclear SKN‐1 proportion was obviously raised from 23.86% to 43.92% (*p* < 0.01), whereas the proportion in cytoplasm was remarkably declined from 43.68% to 18.82% (*p* < 0.01) in LD1 strains when exposed to 3,5‐diCQA (Figure 2A,C). These data demonstrated that the longevity‐promoting benefits of 3,5‐diCQA depended on the activation of SKN‐1.

### 3.3 3,5‐diCQA Scavenged ROS and Improved Oxidative Stress Resistance Through SKN‐1 Regulation

**FIGURE 3:**
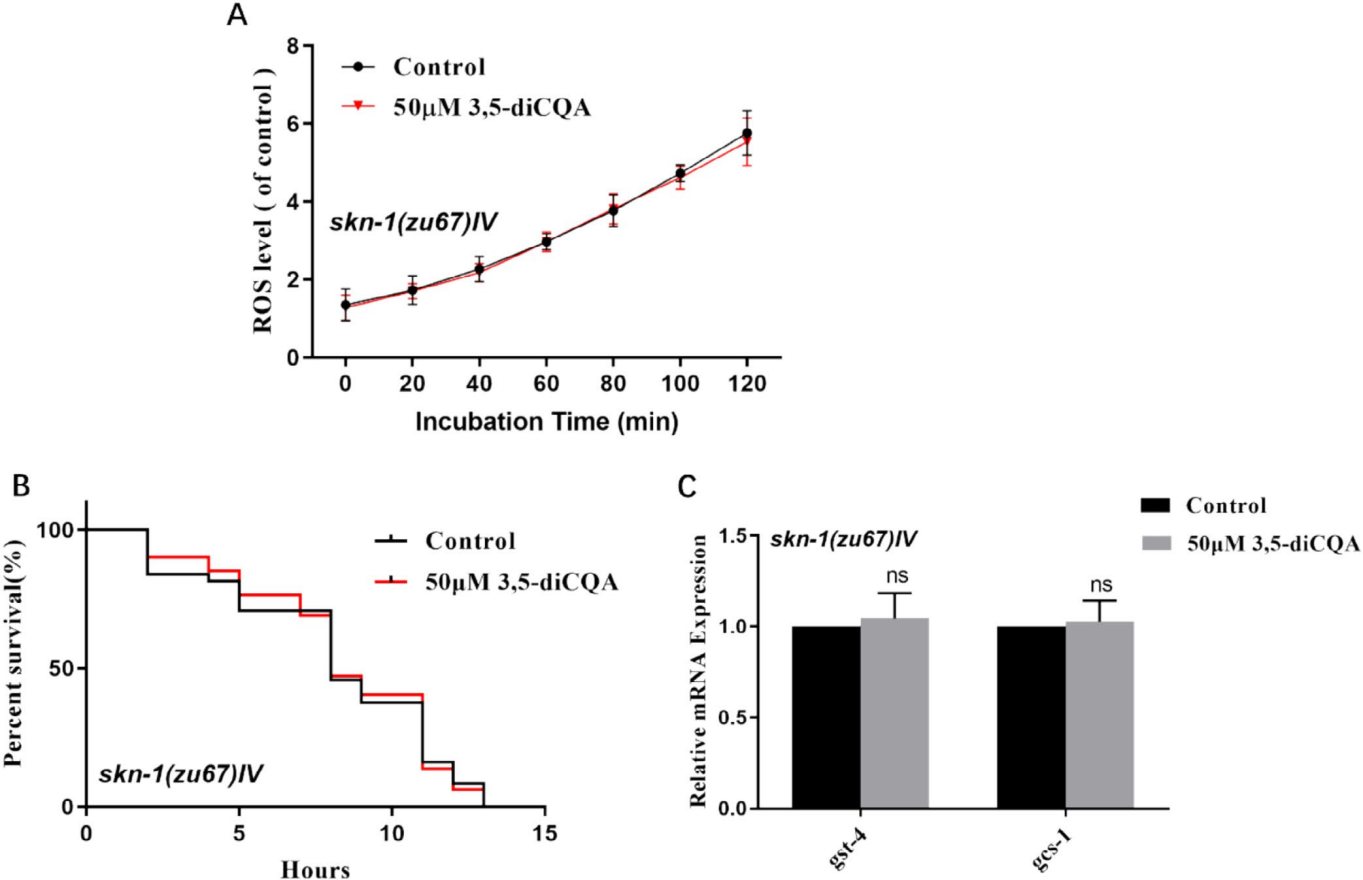
SKN‐1 was essential for 3,5‐diCQA to scavenge ROS and enhance oxidative stress resistance. (A) 3,5‐diCQA failed to decrease the intracellular ROS level of *skn‐1*(*zu67*) mutants. (B) Survival curves of *skn‐1*(*zu67*) mutants under oxidative stress. (C) The mRNA levels of *gst‐4* and *gcs‐1* of *skn‐1*(*zu67*) mutants. Data were displayed as mean ± SD. ns. no significance.

In *C. elegans* , SKN‐1 protects against oxidative stress through the modulation of phase II detoxification, which correlates with the elimination of ROS (Tullet et al. 2008). As displayed in Figure 3A, 3,5‐diCQA was unable to reduce the ROS levels of mutants *skn‐1* (*zu67*) *IV* (*p* > 0.05). Furthermore, the longevity‐promoting effects of 3,5‐diCQA were eliminated in the *skn‐1* null mutants under oxidative stress (Figure 3B, Table S3, *p* > 0.05). Additionally, no significant changes were observed in the expression of *gst‐4* and *gcs‐1* in *skn‐1*‐deficient mutants cultured with 3,5‐diCQA (Figure 3C, *p >* 0.05). Therefore, it implied that SKN‐1 was essential for 3,5‐diCQA to scavenge ROS and enhance oxidative stress resistance.

### 3.4 3,5‐diCQA Delayed Cell Senescence and Decreased the ROS Levels

**FIGURE 4:**
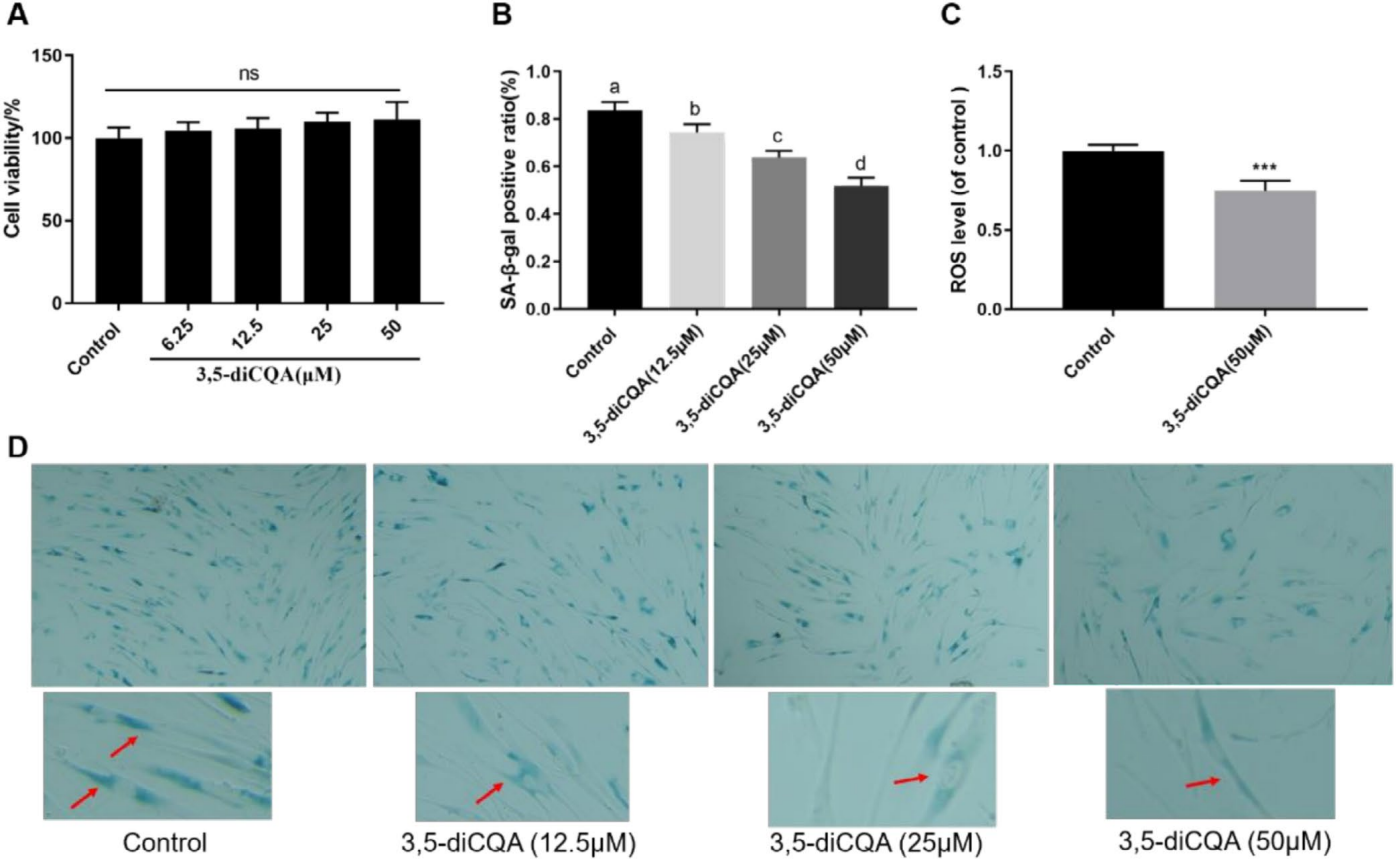
3,5‐diCQA delayed cell senescence and decreased the ROS levels in MRC‐5 cells. (A) Cell viability of MRC‐5 cells treated with 3,5‐diCQA at various concentrations. (B) 3,5‐diCQA decreased the ratio of SA‐β‐gal positive cells. (C) 3,5‐diCQA decreased the ROS level of MRC‐5 cells. (D) SA‐β‐staining images treated in 3,5‐diCQA and control group. Data were displayed as mean ± SD. ***p < 0.001; ns. no significance. Different lowercase letters above the bars presented a significant difference between the two groups.

As a senescence‐associated biomarker, SA‐β‐Gal staining provides an effective approach for evaluating the degree of cellular senescence due to its selective interaction with senescent cells. Increased blue staining intensity indicates a higher number of senescent cells. As displayed in Figure 4A,B,D, 3,5‐diCQA considerably decreased the blue cells number in a dose‐dependent manner with no cytotoxic effects observed. The data revealed that treatment with 3,5‐diCQA significantly lowered the proportion of SA‐β‐gal positive cells, decreasing from 83.8% (control) to 74.3%, 63.9%, and 51.8% at concentrations of 12.5, 25, and 50 μM, respectively. In addition, 3,5‐diCQA treatment resulted in a significant 25.3% reduction in ROS levels in MRC‐5 cells compared to the control group (Figure 4C, *p* < 0.001). Consistent with our observations in *C. elegans* , 3,5‐diCQA exhibited similar anti‐aging effects in human cells by delaying cellular senescence and alleviating oxidative stress.

### 3.5 3,5‐diCQA Activated Nrf2 by Promoting Its Dissociation With Keap1

**FIGURE 5:**
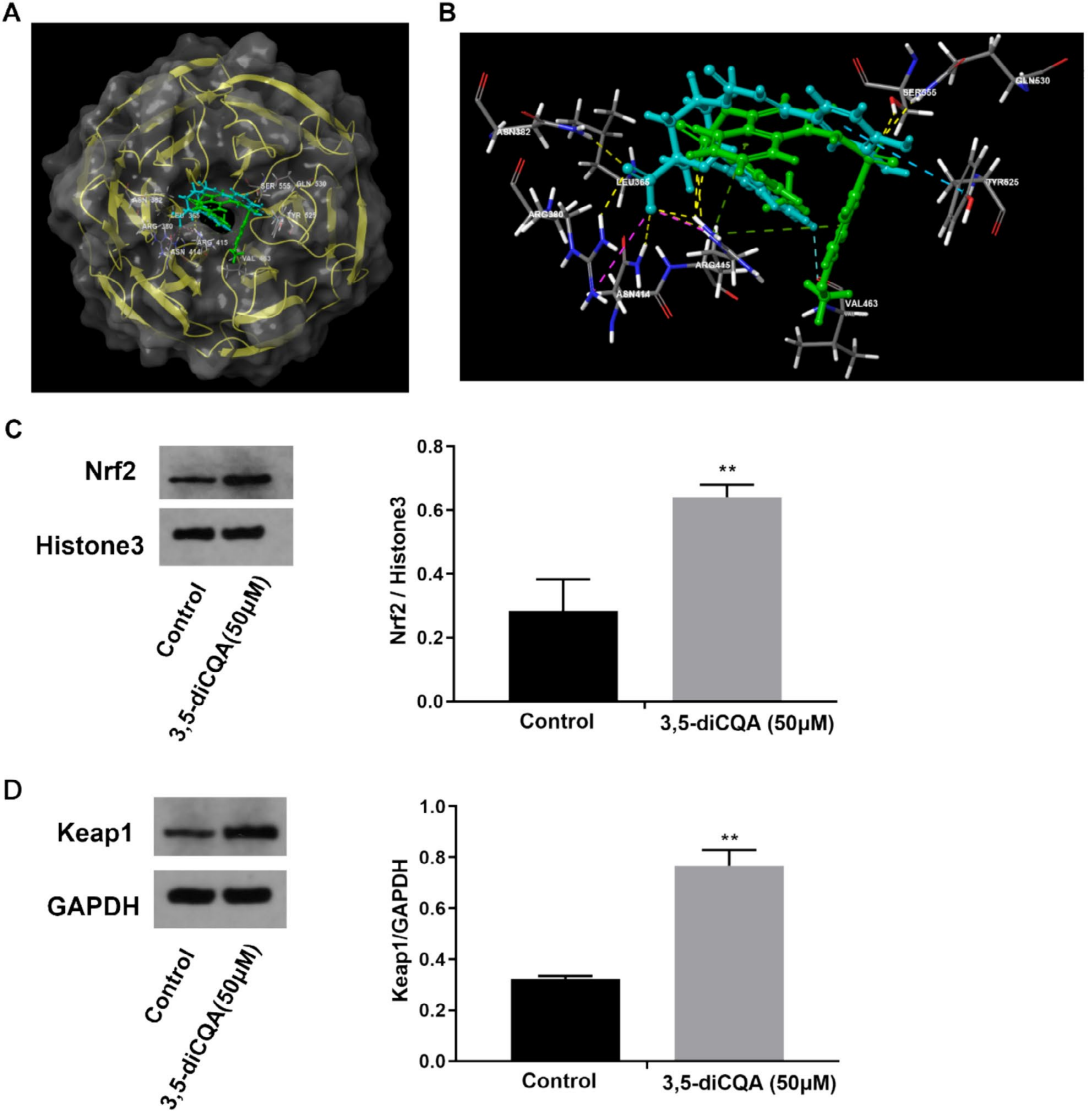
3,5‐diCQA activated Nrf2 by promoting its dissociation with Keap1. (A) Molecular docking of 3,5‐diCQA (light blue) and positive ligand (green) with Keap1. (B) The schematic diagram of 3,5‐diCQA and positive ligand docking with DNA Blind domain of Keap1. (C) Images of Nrf2 in the nucleus and quantification the expression of nuclear protein Nrf2 using Western blotting. (D) Images of Keap1 in the cytoplasm and quantification the expression of cytoplasmic protein Keap1 using Western blotting. Data were displayed as mean ± SD. Statistical significance at **p < 0.01.

To explore the molecular interaction between 3,5‐diCQA and Keap1, we performed molecular docking of 3,5‐diCQA with Keap1. As displayed in Figure 5A, both 3,5‐diCQA and the ligand were stably embedded into the Keap1 active pocket. The docking scores of 3,5‐diCQA and ligand interacting with Keap1 were 7.807 and 5.593, respectively (Table 2), suggesting that 3,5‐diCQA exhibited higher affinity to Keap1 compared to the positive ligand. In addition to forming hydrogen bonds with the side chains of ARG415, LEU365, ASN382, ASN414, VAL463 and ARG380, the carboxyl group of 3,5‐diCQA also engaged in a strong electrostatic interaction with the amino acid residue of ARG415 (Figure 5B). Moreover, the compound 3,5‐diCQA generated π‐π stacking with TYR525 (Figure 5B). These interactions enable 3,5‐diCQA to occupy Nrf2's binding sites within the Keap1 pocket, resulting in the disruption of Keap1‐Nrf2 protein–protein interaction. To further verify the docking results, the expression levels of Keap1 and Nrf2 in MRC‐5 cells treated with 3,5‐diCQA were determined. As expected, 3,5‐diCQA considerably increased the expression of nuclear protein Nrf2 and cytoplasmic protein Keap1 in MRC‐5 cells (Figure 5C,D, *p <* 0.01). Overall, these data demonstrated that by facilitating the dissociation of the Keap1‐Nrf2 complex, 3,5‐diCQA induced the activation of Nrf2, thereby enhancing antioxidant activity and promoting longevity.

**TABLE 2:** Molecular docking statistics of Keap1 with testing compounds.

Compounds	Docking score
Ligand	−5.593
3,5‐diCQA	−7.807

## 4 Discussion

The transcription factor SKN‐1/Nrf2 is recognized to mitigate oxidative stress by regulating the phase II detoxification response, thereby promoting longevity and reducing the risk of aging‐related diseases (Madrigal‐Santillán et al. 2015; Oliveira et al. 2009; Vashi and Patel 2021). 3,5‐diCQA, as a potential activator of SKN‐1/Nrf2, has been reported to extend the survival time and improve healthspan in *C. elegans* (Li et al. 2021). However, the role of SKN‐1/Nrf2 in 3,5‐diCQA‐mediated lifespan extension needs to be further explored. The findings of the current study demonstrated that 3,5‐diCQA alleviated oxidative damage and delayed senescence through activation of the SKN‐1/Nrf2 signaling pathway.

Delaying senescence induced by pharmacologic intervention is often associated with enhanced stress tolerance, particularly against oxidative stress (Denzel et al. 2019; Yang et al. 2018). In *C. elegans* , oxidative stress tolerance exhibits a progressive decline with aging and leads to excessive production of ROS, which in turn further accelerates the aging process (Monickaraj et al. 2013; Sohal and Orr 2012). 3,5‐diCQA, as a common derivative of chlorogenic acid, has been reported to be even more effective in scavenging free radicals than chlorogenic acid and possess antioxidative and UV photoprotective effects in vitro (Baeza et al. 2014; Xu et al. 2012; Fatih et al. 2020). In our study, 3,5‐diCQA was found to considerably decrease the intracellular ROS level in *C. elegans* . Moreover, 3,5‐diCQA markedly increased the survival time of N2 worms under oxidative stress, which was in agreement with a prior study that *Artemisia selengensis Turcz*. leaf extract, rich in 3,5‐diCQA, could obviously reduce ROS level and improve the oxidative stress resistance of *C. elegans* (Li et al. 2022). 3,5‐diCQA, as a natural antioxidant, has the potential to either directly scavenge ROS or indirectly mediate stress‐related signaling pathway. Nevertheless, it is plausible that the protective benefits against oxidative stress in vivo could contribute to the longevity‐promoting effect of 3,5‐diCQA.

Upon its activation by nuclear translocation, SKN‐1, the functional ortholog of mammalian Nrf2, enhances stress tolerance and delays aging (An and Blackwell 2003; Shen et al. 2018). The expression of SKN‐1 in the intestine regulates its stress resistance effect, as SKN‐1 localizes to nuclei and induces the activation of oxidative stress response genes (Bishop and Guarente 2007; Li et al. 2017). Mutation of SKN‐1 causes decreased resistance to oxidative stress and shortened survival time, whereas overexpression of SKN‐1 leads to extended lifespan and enhanced oxidative stress resistance (Turner et al. 2024; Ye et al. 2019; Wan et al. 2020). In the present study, 3,5‐diCQA was found to facilitate the translocation of SKN‐1 into the nucleus and upregulate the expression of *skn‐1*, indicating that 3,5‐diCQA might act as an activator of SKN‐1. This finding was corroborated by the fact that the expression levels of *gst‐4* and *gcs‐1* were significantly increased in 3,5‐diCQA‐treated nematodes. As the *C. elegans* orthologs of human glutathione S‐transferase and glutamate‐cysteine ligase catalytic subunit, *gst‐4* and *gcs‐1* facilitate the elimination of free radicals. In addition, the expression levels of GST‐4::GFP and GCS‐1::GFP were also upregulated in the nematodes cultured with 3,5‐diCQA. Our results were in accordance with a prior study showing that chlorogenic acid, a homologue of 3,5‐diCQA, activated transcription factor SKN‐1 to extend lifespan (Zheng et al. 2017). Furthermore, the ROS‐scavenging and lifespan‐extending effects of 3,5‐diCQA were completely eliminated in the *skn‐1‐deficient* mutants. Similarly, 3,5‐diCQA failed to upregulate the expressions of *gst‐4* and *gcs‐1* in the *skn‐1* null mutants. These findings illustrated that 3,5‐diCQA enhanced oxidative stress resistance and decreased ROS levels in a *skn‐1‐*dependent manner.

The anti‐aging effect of 3,5‐diCQA was further confirmed in the MRC‐5 cells model. The results showed that 3,5‐diCQA considerably reduced the ROS level and delayed senescence of MRC‐5 cells. It is well‐documented that the transcription factor Nrf2 plays a pivotal role in defense against oxidative stress and in mediating the expression of phase II detoxifying enzymes (Kobayashi and Yamamoto 2005; Zatorski et al. 2015). Under normal physiological conditions, Nrf2 is retained in the cytoplasm due to its binding with the Keap1 protein. However, certain small‐molecule active compounds could trigger the disassembly of the Nrf2‐Keap1 complex, allowing Nrf2 to release and translocate into the nucleus, thereby activating the antioxidant signaling pathway (Bello and Morales‐González 2017). Molecular docking results demonstrated that 3,5‐diCQA and a positive ligand were effectively embedded into the Keap1 binding pocket, indicating that 3,5‐diCQA could potentially act as a Nrf2 activator, thereby facilitating the nuclear translocation of Nrf2. As expected, 3,5‐diCQA treatment increased the expression levels of nuclear protein Nrf2 and cytoplasmic protein Keap1 in MRC‐5 cells, suggesting that 3,5‐diCQA activated Nrf2 by inducing its dissociation from the Keap1 complex. This corroborated a previous finding that 3,5‐diCQA ameliorated oxidative stress in Caco‐2 cells by activating the Nrf2‐Keap1 signaling pathway (Liang and Kitts 2018). Notably, there is no homologous protein of Keap1 in *C. elegans* (Lo et al. 2017). However, combining with the results that 3,5‐diCQA prolonged the survival time of worms by activating the Nrf2 ortholog protein SKN‐1, we speculated that 3,5‐diCQA was able to activate SKN‐1/Nrf2 via both Keap‐1‐dependent and Keap‐1‐independent manners, highlighting its comprehensive efficacy in SKN‐1/Nrf2 activation.

3,5‐diCQA is considered to be a natural substance and may be safely used as a medication, food, or a cosmetic. It has been reported that 3,5‐diCQA can be used in the prevention and treatment of a muscle disorder, or improvement in muscle function (Hwang et al. 2021). In addition, 3,5‐diCQA has been documented as the primary phenolic compound in Gongju, demonstrating efficacy in mitigating liver injury and positioning it as a promising candidate for hepatoprotective diets (Ma et al. 2024).

In summary, our findings illustrate that 3,5‐diCQA delays senescence and enhances oxidative stress resistance by activating the SKN‐1/Nrf2 signaling pathway. Moreover, 3,5‐diCQA prolonged the lifespan and reduced the ROS level of worms in a *skn‐1*‐dependent manner. Additionally, 3,5‐diCQA facilitated Nrf2 activation through direct interference with the Keap1‐Nrf2 complex. Collectively, this research uncovered new evidence demonstrating that 3,5‐diCQA functions as a novel SKN‐1/Nrf2 activator to delay aging and attenuate oxidative stress.

## Author Contributions

**Rong Li:** conceptualization (equal), writing – original draft, data curation (equal), writing – review and editing (equal), funding acquistion. **Mingfang Tao:** conceptualization (equal), data curation (equal), formal analysis (equal). **Jinzhan Yuan:** writing – review and editing (equal). **Yechuan Huang:** data curation (equal), formal analysis (equal), investigation (equal). **Tingting Xu:** formal analysis (equal), methodology (equal). **Xiaoyun Xu:** conceptualization (equal), methodology (equal).

## Funding

This work was supported by Doctoral Research Initiation Fund of Jingchu University of Technology, YY202403; Jingmen Youth Talent Science and Technology Program Project, 2025QNRC01.

## Conflicts of Interest

The authors declare no conflicts of interest.

## Supporting information


**Table S1:** Primers used in this research.
**Table S2:** The effect of 3,5‐diCQA on the lifespan of EU1 worms.
**Table S3:** The effect of 3,5‐diCQA on the oxidative stress resistance of EU1 worms.

## Data Availability

Data will be made available on request.
