# Supplementary material for: 3,5‐Dicaffeoylquinic Acid Delayed Aging and Promoted Oxidative Stress Tolerance via Activation of the SKN‐1/Nrf2 Signaling Pathway
Source: Food Sci Nutr. 2026 Feb 17;14(2):e71532. doi: 10.1002/fsn3.71532 (PMC12910240; doi:10.1002/fsn3.71532)
Supplement: Supplementary file 1 — Table S1: Primers used in this research. Table S2: The effect of 3,5‐diCQA on the lifespan of EU1 worms. Table S3: The effect of 3,5‐diCQA on the oxidative stress resistance of EU1 worms. [file FSN3-14-e71532-s001.docx]

**3,5-Dicaffeoylquinic acid delayed aging and promoted oxidative stress tolerance via activation of the SKN-1/Nrf2 signaling pathway**

**Rong Li^1*^, Mingfang Tao^2^, Jinzhan Yuan^3^,Yechuan Huang^1^, Tingting Xu^4^, Xiaoyun Xu^4^**

^1^ Characteristic food function mining and comprehensive utilization research center, Jingchu University of Technology, Jingmen, China.

^2^ Hubei Key Laboratory of Nutritional Quality and Safety of Agro-products, Institute of Agricultural Quality Standards and Detection Technology, Hubei Academy of Agricultural Sciences, Wuhan, China.

^3^ College of Plant Science and Technology, Huazhong Agricultural University, Wuhan, China.

^4^ Key Laboratory of Environment Correlative Dietology (Ministry of Education), College of Food Science and Technology, Huazhong Agricultural University, Wuhan, China.

*** Corresponding Author**

Rong Li : lirong2022@jcut.edu.cn;

**Supplementary Table S1**. Primers used in this research.

| Gene | Primers |
| --- | --- |
|  |  |
| *act-1* | F: CATGAAGATCAAGATCATCGCC |
|  | R: GTGACGATGGTTTTGAACTTGT |
| *skn-1* | F: CTTCAGGACGTCAACAGCAG |
|  | R: GATTCCGAAGAGAGGCGAGA |
| *gst-4* | F: TCTTGCTGAGCCAATCCGTA |
|  | R: AATGGGAAGCTGGCCAAATG |
| *gcs-1* | F: GTCGATGAAGCCAGATGGTTGT |
|  | R: CGATCGTCGACACTTGCACTAA |

**Supplementary Table S2.** The effect of 3,5-diCQA on the lifespan of EU1 worms

| **Treatments** | **Mean lifespan**  **(days ± SEM)** | **Percentage Change** | **Number of worms** | ***p* value** |
| --- | --- | --- | --- | --- |
| Control | 14.53 ± 0.52 | - | 110 | - |
| 50 μM 3,5-diCQA | 15.15 ± 0.60 | 4.27% | 102 | 0.2772 |

**Supplementary Table S3.** The effect of 3,5-diCQA on the oxidative stress resistance of EU1 worms

| **Treatments** | **Mean lifespan**  **(hours ± SEM)** | **Percentage Change** | **Number of worms** | ***p* value** |
| --- | --- | --- | --- | --- |
| Control | 8.07 ± 0.27 | - | 168 | - |
| 50 μM 3,5-diCQA | 8.35 ± 0.25 | 3.47% | 162 | 0.9049 |
